# Optimization of neuronal cultures from rat superior cervical ganglia for dual patch recording

**DOI:** 10.1038/srep14455

**Published:** 2015-09-24

**Authors:** Julien Amendola, Norah Boumedine, Marion Sangiardi, Oussama El Far

**Affiliations:** 1 INSERM, UMR_S 1072, Marseille, France; 2 Aix-Marseille Université, Marseille, France

## Abstract

Superior cervical ganglion neurons (SCGN) are often used to investigate neurotransmitter release mechanisms. In this study, we optimized the dissociation and culture conditions of rat SCGN cultures for dual patch clamp recordings. Two weeks *in vitro* are sufficient to achieve a significant CNTF-induced cholinergic switch and to develop mature and healthy neuronal profiles suited for detailed patch clamp analysis. One single pup provides sufficient material to prepare what was formerly obtained from 12 to 15 animals. The suitability of these cultures to study neurotransmitter release mechanisms was validated by presynaptically perturbing the interaction of the v-SNARE VAMP2 with the vesicular V-ATPase V0c subunit.

Adult sympathetic rat superior cervical ganglion neurons (SCGN) were one of the first neuronal systems used to investigate synaptic neurotransmission mechanisms^1^. These neurons have been a very useful system, as illustrated by an abundant literature, especially to study intracellular pathways of ion channel regulation^2^,^3^ and ion channel signalling mediated via muscarinic receptors^4^,^5^,^6^. SCGNs have also been exploited to develop a dissociated neuronal SCG culture model to study synaptic neurotransmission by perturbing presynaptic protein-protein interactions through injection of interfering molecules^7^,^8^,^9^. *In vivo*, SCG neurons are principally adrenergic with a few cells displaying a cholinergic phenotype^10^. Depending on culture conditions, SCGN can switch phenotype *in vitro* and develop cholinergic transmission^10^,^11^,^12^,^13^,^14^. This phenotypic switch has been made use of to follow up membrane currents and voltage changes associated with acetylcholine release using sharp electrode intracellular recording in studying neurotransmission mechanisms.

A detailed characterization of the presynaptic mechanisms SNARE-mediated neurotransmitter release has been performed using SCGNs^7^. This was facilitated by the very large size of these neurons, allowing direct injection in the soma of interfering molecules such as peptides, fusion proteins and even intra-nuclear injection of cDNA^15^ coding for specific proteins. In this study, we present a protocol to prepare up to six SCGN coverslips from one single ganglion and therefore significantly reduce the number of animals used. In addition, our dissociation and culture conditions allow dual patch-clamp recording and monitoring of cholinergic neurotransmission in as early as two week-old dissociated cultures, rather than the commonly used six to eight week-old cultures^7^.

## Results

Several SCG dissociation protocols have been reported by different laboratories in order to prepare SCGN cultures for multiple purposes. For synaptic transmission studies, neuronal membrane access was often not optimal for patch clamp recording and usually one ganglion was plated on one single coverslip. Collagenase-based dissociation is inefficient and is followed by harsh mechanical trituration that can damage neurons and limit their survival (one ganglion/one coverslip). We used sequential treatment by collagenase and trypsin. This allowed better neuronal extraction with limited mechanical manipulation and therefore improved neuronal viability. Out of two ganglia (one P6–7 day-old pup), we were able to prepare twelve 18 mm coverslips with 538 ± 63 neurons per coverslip, enough to record several pairs of neurons on each coverslip.

### Morphology and electrical properties of neurons during *in vitro* development

**Figure 1:**
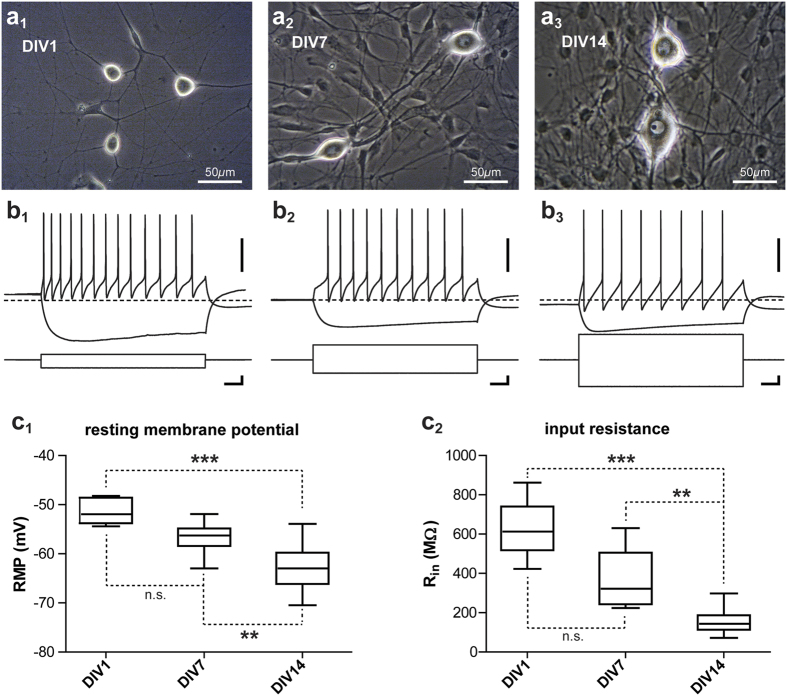
Morphology and electrical properties development of cultured SCGN. (**a**) Photomicrographs of SCGNs and surrounding non-neuronal cells at DIV1, DIV7 and DIV14 respectively for a_1_, a_2_ and a_3_. Neurons are easily identifiable by their big bright somata. Note the increase in neuronal soma size during this period. (**b**) Electrical profiles of DIV1, DIV7 and DIV14 neurons (for a_1_, a_2_ and a_3_ respectively) submitted to hyperpolarizing and depolarizing steps of current, scale bar 40 mV, 100 pA and 200 ms for each. The dashed-line represents −60 mV. (**c**) Evolution of SCGN resting membrane potential (c_1_) and input resistance (c_2_) from DIV1 to DIV14. Each box is delimited by the first and third quartiles and is crossed by the median value. The ends of the bars are the lower and the minimal values. The Kruskal-Wallis test was used to compare the evolution of input resistance and resting membrane potential over the 3 age groups as these parameters were not normally distributed in all groups. Then Dunn’s multiple comparison test was used as a post-test to compare all the groups between themselves.**P < 0.01, ***P <0 .001, n.s., Not Significant.

Figure 1 illustrates, at three different stages, the morphological and electrical profiles of SCG neurons maintained in culture. At DIV 1, (Fig. 1a) neuronal cells could be distinguished by their bright soma and clear neuritic processes. Compared to neurons, non-neuronal cells were flatter and less light-refractive (Fig. 1a_1_,b_1_,c_1_). At DIV7, an increase in neuronal soma size and also a higher density of non-neuronal cells was noted (Fig. 1b1). In this system, neurons develop on a layer of non-neuronal cells. The adjunction of antimitotic drugs at this stage limited non-neuronal proliferation but did not affect the increase in neuronal soma size as observed at DIV14 (Fig. 1c_1_). Although we have not performed a systematic morphological analysis, big neuronal soma exceeding 40 *μ*m in diameter were commonly observed as illustrated in Fig. 1c.

Between DIV1 and DIV14, resting membrane potential (RMP) and input resistance (Rin) showed the classical evolution of neuronal properties usually observed during maturation^16^,^17^,^18^,^19^. RMP progressed to more hyperpolarized values with the age of cells, with an average value of −51.47 ± 2.53  mV at DIV1–2 (n = 6), −56.63 ± 2.94 mV at DIV 7 (n = 10) and −62.96 ± 4.18 mV (n = 29) at DIV 14–17 (Fig. 1d, P < 0.001, Kruskal-Wallis test). This is illustrated in Fig. 1a2,b2, b2 where, before the current steps, the membrane potential was respectively for DIV 1 and DIV 14 above and under the −60 mv dashed line.

Input resistances were on average 626.7 ± 148.7 MΩ (n = 6), 361.9 ± 141 MΩ (n = 10) and 154.6 ± 54.36 MΩ respectively at DIV1–2, DIV 7 and DIV 14–17 (Fig. 1e, P < 0.001, Kruskal-Wallis test). This decrease of input resistance with age is illustrated by the fact that smaller amplitudes of negative current induced similar or even more pronounced hyperpolarization in younger neurons as seen in Fig. 1b. This decrease of input resistance could be correlated to the increase in neuronal size (Fig. 1a_2_,b_2_,c_2_). This decrease could also support the fact that younger neurons were able to reach similar or higher firing frequencies with smaller positive currents (Fig. 1a_2_,b_2_,c_2_). As illustrated in panel 1b_2_, some neurons showed a delay between the beginning of depolarization and the emission of the first AP. This membrane potential rectification was sometimes observed at DIV 7 and in older cultures, but never at younger stages. This behavior was probably due to the inward rectifying potassium current well described in sympathetic neurons^20^,^21^. In order to verify whether the enzymatic treatments as well as the plating dilution affected neuronal properties, we measured RMP, Rin, AP threshold & amplitude, as well as AP half duration in DIV 14–17 neurons. As presented in Table 1, these values indicated that these neurons had healthy profiles.

### Growth in ciliary neurotrophic factor (CNTF) increased neuronal connectivity

**Figure 2:**
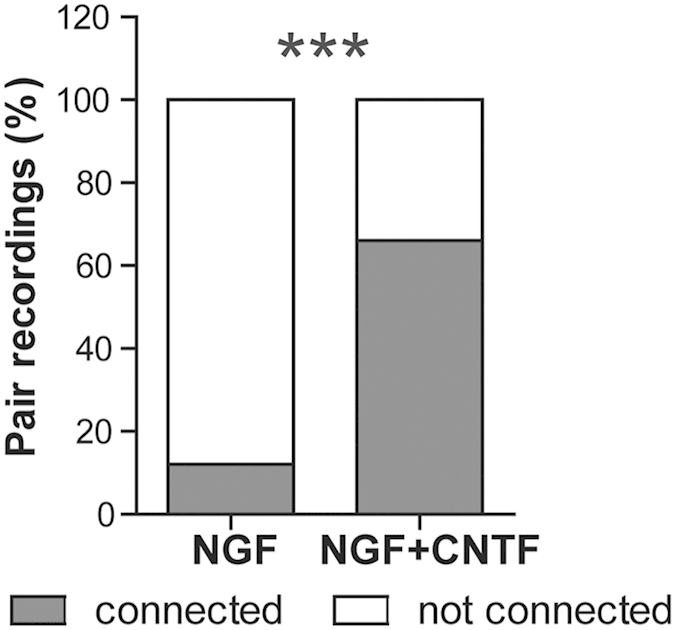
CNTF increases the connectivity between cultured SCGNs. Stacked bars plots showing the percentage of pair recordings with connected and unconnected neurons in the presence or absence of CNTF in the culture medium. When CNTF was added to the culture media at DIV2, the neuronal connectivity tested by pair recording at DIV14–17 increased from 12 to 66%. The Fisher exact test was used to compare the proportion of connected neurons in the 2 groups. Statistics were done on numerical values (7 out of 57 connected pairs for NGF vs 38 out of 58 when CNTF was added) and not the percentage as presented on the plot.

Previous studies estimated the degree of cholinergic connectivity in SCGN cultures and showed that over 60% of the neurons were connected in conditions containing NGF as the sole extraneously added growth factor^7^. By measuring the enzymatic activity and the expression levels of choline acetyltransferase, early studies reported that CNTF increased the *in vitro* cholinergic differentiation of SCGNs^22^. Autaptic SCG cultures were successfully cultured in the presence of CNTF^23^ and a more recent study using patch clamp recording on single SCG neurons showed that CNTF enhanced nicotinic synaptic transmission^24^. The absence of trypsin in the enzymatically assisted dissociation of SCGs appeared to be a major difference between the protocols using NGF alone and those including CNTF. Therefore, using our dissociation conditions and plating dilution, we compared the effect on cholinergic connectivity, of culture media containing NGF alone or supplemented with saturating concentrations of CNTF at DIV 14–17. When the culture media was supplemented with NGF as the only growth factor, connection probability was as low as 12% (7 out of 57 tested pairs, Fig. 2). However, the addition of CNTF to the culture medium from DIV 2 (see materials and methods) significantly increased the neuronal connectivity rate from 12 to 66% (Fisher exact Test P < 0.001) (38 out of 58 recorded pairs) (Fig. 2).

### Cholinergic synaptic currents and dual recordings from connected neuronal pairs

**Figure 3:**
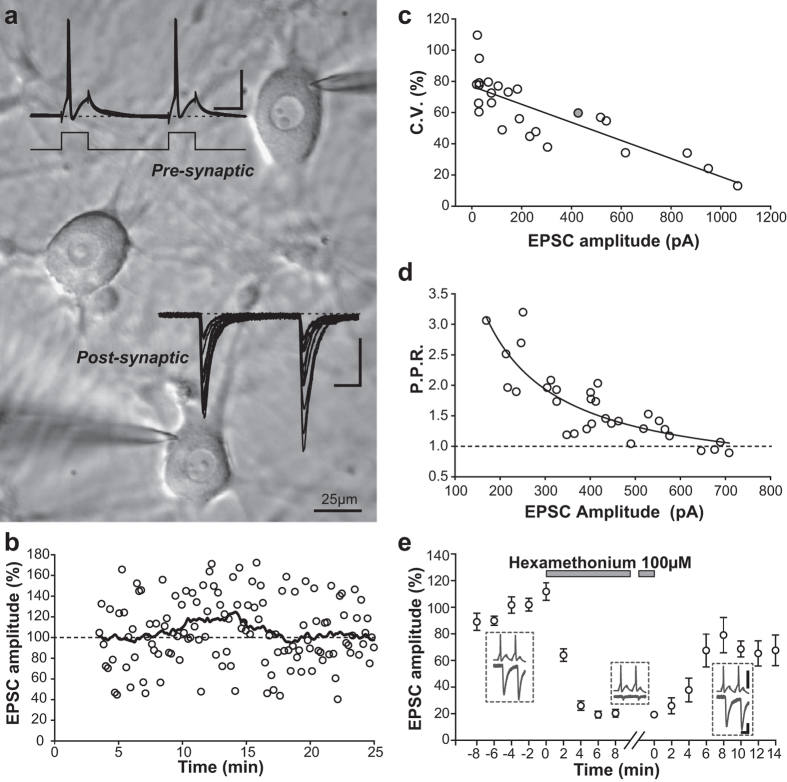
Paired recordings of connected neurons and the neurotransmitter nature of synaptic connections. (**a**) Photomicrograph (magnification 40×) of patched SCG neurons. Recording microelectrodes are visible on connected neurons in the upper and lower part of the image. EPSCs (post) evoked by individual spikes in the presynaptic neuron (pre) were recorded and superimposed on the photomicrograph (twelve traces are represented). Scale bars 40 mV, 20 ms and 200 pA. Dashed lines represent −60 mV and 0 pA respectively for the pre-synaptic and the post-synaptic element. (**b**) Normalized amplitude of the EPSC monitored over time for a typical neuronal pair. Each empty circle represents the amplitude of a single EPSC after a single AP and illustrates the trial-to-trial variation in the response. Only the amplitude of the first action potential was plotted over time. The black line is the smoothed running average (every two minutes) of the normalized EPSC amplitude. For all the duration of the recording, this average value stayed close to the base line level (100%) represented by the dashed-line. (**c**) Plot of the coefficient of variation (CV) as a function of EPSCs amplitudes for the 26 neuronal pairs recapitulated in Table 2. The relation well fitted with a linear regression y = a*x + y_0_ where a = −0.0579 and y_0 _= 76,9 (r^2 ^= 0.66). (**d**) Plot of the amplitude of the first EPSC for the gray neuronal pair shown in (**c**) as a function of the paired-pulse ratio. Paired-pulse facilitation was observed for EPSCs below 300 pA while paired-pulse depression seem to take place for EPSCs larger than 600 pA. This plot was well fitted by the equation y = (a/x) + y_0_ where a = 459 and y_0_ = 0.4 (r^2 ^= 0.71). (**e**) Normalized EPSC amplitudes for the first action potential monitored over time for five connected pairs of neurons before, during and after Hexamethonium application. Average response and s.e.m. are represented every 2 minutes. Insets before, during and after hexamethonium application represent the corresponding average traces over 1 minute intervals (6 traces) for double pulse stimulations. Scale bars are 40 mV, 20 pA and 20 ms. The plot shows an almost complete block by hexamethonium and a partial recovery of the response during its washout.

Based on a previous report, the number of mono-synaptically connected SCG neurons increases until approximatively DIV 14 7. Therefore, we performed our neuronal pair recordings in the 3^rd^ post-culture week (DIV 14–17). Figure 3a illustrates an example of pair-recording of mono-synaptically connected neurons. The plot of EPSC amplitude over time is shown in Fig. 3b. Despite an important variability in EPSC amplitudes and several failures, this figure illustrates the fact that our experimental conditions allow stable recording for several tens of minutes. During this time window, parameters such as input resistance, access resistance and injected current in both the pre- and postsynaptic neurons were also monitored and remained constant during the recording (not shown). Several EPSC parameters such as amplitude, latency, kinetics and variability of these inward currents are given in Table 2. Analysis of the 26 neuronal pairs mentioned in Table 2 showed a linear correlation between EPSCs amplitudes and the coefficient of variation (CV; Spearman Rank Order Correlation: r^2 ^= 0.695 and P < 0.001, n = 26; Fig. 3c). The CV corresponds to the standard deviation of action-potential-dependent EPSCs divided by their mean amplitude. This measure describes the EPSCs’ trial-to-trial amplitude fluctuation. A decrease in the coefficient of variation is typically associated with an increase in transmitter release probability. This analysis shows that EPSCs amplitudes increase correlates with an increase of release probability. For individual recordings, the PPR values were plotted as a function of EPSCs amplitudes. Figure 3d shows a plot illustrating EPSC amplitudes as a function of PPR for the neuronal pair highlighted with a grey circle in Fig. 3c. Paired-pulse facilitation was observed for EPSCs below 300 pA while paired-pulse depression seem to take place for EPSCs larger than 600 pA. This correlation is similar to what was observed previously in central CA3-CA3 synapses^25^.

In order to characterize the neurotransmitter identity of our SCGN synaptic connections and verify whether the expected cholinergic phenotypic switch is taking place, we used hexamethonium, a prototypical cholinergic ganglionic blocker. At DIV 14–17, the nearly exclusive cholinergic nature of neuronal connections was confirmed by the fact that 100 *μ*M hexamethonium almost completely and reversibly blocked recorded EPSCs (n = 5) (Fig. 3e). Insets before, during and after hexamethonium application represent the corresponding average traces for double pulse stimulations. PPR analysis, before and after hexamethonium treatment indicated no significant change (Not shown; P = 0.125 with Wilcoxon matched pairs signed-rank test) corroborating thus that the effect of hexamethonium is clearly postsynaptic.

### Suitability to study neurotransmitter release mechanisms

**Figure 4:**
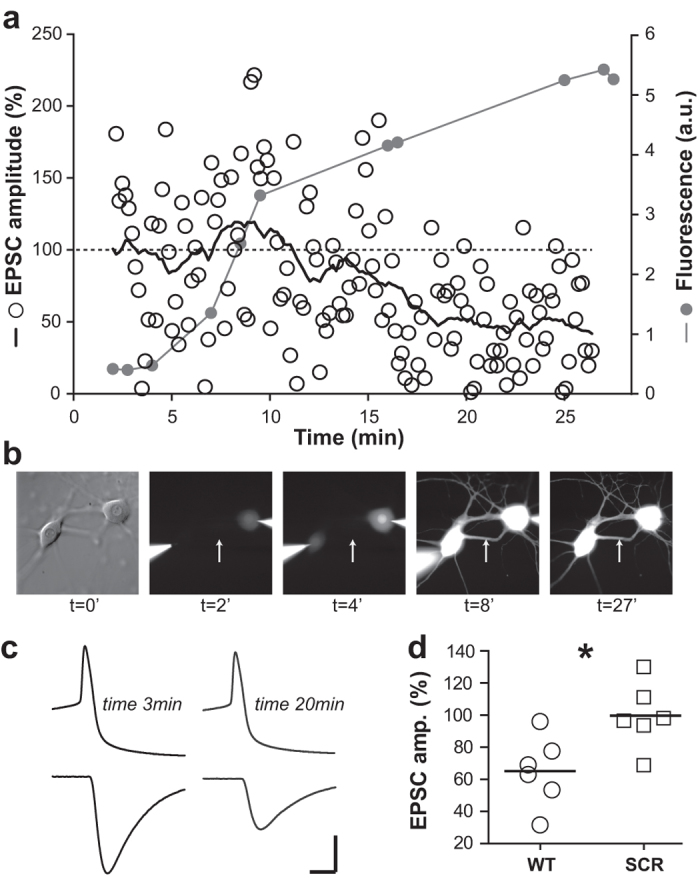
Diffusion rate and inhibitory effect of peptide injection. (**a**) Normalized EPSC amplitude monitored over time for the pair of neurons shown in b. Empty circles represent the amplitude of single EPSCs and the black line the smoothed running average (every two minutes) of these values. The dashed line represents the base line level (100%) of EPSC amplitude. Grey lines and circles represent the level of fluorescence measured over time on cell processes. Note that the intensity of this signal reaches approximatively half of its maximal value around 8–10 minutes exactly when the amplitude of the EPSC starts to decrease. (**b**) Photomicrographs of the recorded pair of neurons corresponding to the data shown in (**a**). Note the progressive increase of fluorescence intensity in the soma (t = 4′) and then in neurites (t = 8′ and 27′). The white arrow represents the position where the intensity of fluorescence illustrated in (**a**) was measured. (**c**) Pre- and post-synaptic signals at fast time-scale, before (3 min) and during L3.4 peptide injection (20 min). Scale bars are 4 ms, 40 pA, 40 mV. Note the decrease of EPSC amplitude while the AP shape is not affected by the peptide. An average of 6 traces is illustrated for each time. (**d**) Normalized EPSC amplitudes ± s.e.m. at 20–25 min in the presence of L3.4 peptide (WT) 65.2 +/− 8.9% or its scrambled form (SCR) 99.8 +/− 8.3 ; *P = 0.026 (Mann-Whitney U test). The horizontal bars represent the mean for each group.

We have used the SCGN culture system to measure perturbations in neurotransmitter release mechanisms^26^. In these experiments, the protocol of SCGN preparation used the ratio of 1 ganglion/coverslip, only collagenase for ganglia dissociation, did not include CNTF and neurons were recorded after 6 weeks *in vitro*. In order to verify if under the new culture conditions, SCGN can still be used efficiently to investigate perturbations in neurotransmitter release mechanisms, we tested the influence of presynaptic injection of the loop 3.4 of the ATP-V0c subunit. ATP-V0c is a component of the synaptic vesicle V-ATPase that directly interacts with the juxta-membrane domain of the v-SNARE synaptobrevin and modulates neurotransmitter release. A synthetic peptide corresponding to the loop 3.4 (L3.4) of the V0c subunit drastically inhibited release when present in the recording pipette (Fig. 4), rather than being injected using a third injection microelectrode as previously reported^26^. In the example illustrated in Fig. 4a, EPSC amplitude started to decrease almost 10 min after breaking the seal and stabilized to 47% of the initial amplitude after 20–25 minutes. Figure 4c illustrates pre- and post-synaptic signals at fast time-scale, before (3 min) and during L3.4 peptide injection (20 min). On average when L3.4 peptide was injected through the recording pipette, EPSC amplitudes stabilized to 65.2 ± 8.9% (n = 6) of the control amplitude after 20–25 minutes (Fig. 4d). In contrast, no reduction in amplitude occurred when the presynaptic pipette contained the scrambled L3.4 s peptide (99.8% ± 8.3%, n = 6, Fig. 4d); p = 0.026 Mann-Whitney U test.

In order to investigate whether the diffusion rate of perfused drugs correlates with the observed inhibition kinetics, we added Alexa fluor 594 to the recording pipettes and monitored its diffusion by video-microscopy. As shown in Fig. 4b, perfused Alexa fluor 594 was present in the distal part of the cells 8 minutes after breaking the seal and going into the whole cell configuration, almost coinciding with the onset of inhibition of neurotransmitter release (Fig. 4a).

## Discussion

Several laboratories have already used SCGN cultures to study different biological issues from ion channel function to neurotransmitter release mechanisms. Multiple dissociation and cell culture protocols have been employed successfully. The protocols we have compared in this study either used collagenase alone or a mixture of collagenase and trypsin. A common feature between the studies with CNTF, was the use of trypsin during the dissociation procedure. CNTF is a multifunctional neurotrophic factor that was originally purified from the chick ciliary ganglia. It plays a role in glial and neuronal survival and promotes cellular differentiation in the central and peripheral nervous system^27^. Saadat *et al.*^22^ has previously shown *in vitro* that CNTF plays an important role in the cholinergic switch of SCGN^22^. This noradrenergic to cholinergic switch takes place through an increase in choline acetyltransferase expression levels. Again, this study was made on SCGNs that had been treated with trypsin. In the protocol we used initially to prepare SCGN cultures to study neurotransmitter release mechanisms, we plated one single ganglion per coverslip and did not use trypsin or CNTF. In an attempt to reduce the number of animals required, we modified our ganglia dissociation protocol to include trypsin digestion. This resulted in increased neuronal yield, producing up to 12 coverslips per rat. However, the neuronal connectivity rate dropped drastically in comparison to the previous protocol^7^. The subsequent addition of saturating concentrations of CNTF allowed recovery and 66% of neurons became cholinergically connected at DIV14–17 (Figs 2 and 3). The origin of the important variability in EPSC amplitude was analysed by plotting EPSC amplitudes as a function of coefficient of variation (Fig. 3c) as well as a function of PPR (Fig. 3d). In each case, a significant anti-correlation was found; this variability clearly correlates with release probability fluctuations. The interpretation of the effect of trypsin is not entirely clear. However, it is likely that the addition of trypsin affects the viability of non-neuronal cells, secreting CNTF or a functionally related growth factor that induces the cholinergic phenotypic switch *in vitro*. It is noteworthy that the mean excitatory post-synaptic current amplitude was relatively high (267.3 pA ± 60.9 pA, n = 26). This large amplitude was in accordance with the large average amplitude of the mean post synaptic potential (20 ± 6.8 mV, n = 10) formerly described^7^ and significantly more elevated than EPSCs usually observed in dissociated hippocampal or slice cultures^28^,^29^ which facilitates measurements as the signal to noise ratio is high.

Finally, our new culture conditions allow recordings of connected neuronal pairs as early as DIV 15–17, in comparison to our former conditions which required long maturation (up to 8 weeks). In addition, rather than positioning a third injection pipette presynaptically, we used the presynaptic recording pipette, to perfuse agents previously shown to perturb exocytosis. The rate of inhibition of neurotransmitter release (Fig. 4) that was achieved was very similar to that in our earlier report^26^.

Therefore, the new protocol for SCG cultures limits animal usage and is well suited for studying neurotransmitter release mechanisms.

## Methods

### Dissection

The experiments proposed in our work are based on the use of organs and tissues collected from dead animals, after euthanasia. No specific validation of experimental protocols is required by French law as death is given to animals for the scientific uses of their organs and tissues: the modalities of this rule are specified by the French Orders published in the “Official Journal” of the French Republic on February 7th 2013 (orders AGRG1231951D; AGRG12400332A; AGRG1238753A; AGRG1238729A; AGRG1238767A; AGRG1238724A). In this research, the requirements of Directive 2010/63/EU (which repealed Directive 86/609/EEC from 1st January 2013) of the European Parliament and of the Council of 22 September 2010 on the protection of animals used for scientific purposes as well as French national law implementing this directive (orders AGRG1231951D; AGRG12400332A; AGRG1238753A; AGRG1238729A; AGRG1238767A; AGRG1238724A) and regulating researches on animals are fully respected. 6–7 day old postnatal rat was anesthetized by isoflurane before decapitation and superior cervical ganglia (SCG) were dissected under a stereoscopic microscope (Nikon SMZ-10A, 8×−40× magnification). SCGs are easily identifiable just underneath and medial to each carotid artery, close to the bifurcation into internal and external carotids. SCGs were removed and put in a dish containing L15 medium (Life Technologies™) with 5% of penicillin-streptomycin (PS; Life Technologies™) on ice. Surrounding attached tissues were removed and ganglia were then unsheathed with fine forceps (Dumont #55). Finally, using fine scissors, small notches were made in the transversal plane of each ganglion in order to increase the efficiency of enzymatic treatment.

### Preparation of SCGN cultures

Ganglia were incubated for 12–15 minutes at 37 °C in 1 mL L15 media containing 0.1 U/mL collagenase type I (Worthington, Biochemical Corporation). L15 was then completely removed and ganglia incubated for 25–30 minutes at 37 °C in 5 mL buffered Ca^2+^ and Mg^2+^-free HBSS (Life Technologies™) buffered with 10 mM Hepes containing 0.05% trypsin (Sigma-Aldrich®). Trypsin was removed and ganglia were placed in 1 mL MEM-based growth medium containing 2%fetal calf serum ((Life Technologies™)), 3% horse serum (Life Technologies™), 1% PS, 1% Glutamax (Life Technologies™) and 40 ng nerve growth factor (NGF; Sigma-Aldrich® or Euromedex group). At this stage using a 1 mL pipette tip (1.2 mm tip diameter), ganglia were triturated up and down 20–30 times to dissociate cells. Then a smaller pipette tip (0.8 mm tip diameter) was used to triturate cells up and down 15–20 times. At this point, the cloudy suspension of cells was put on ice for 2  minutes so that undissociated debris settled to the bottom. Medium from the top with suspended cell was carefully collected and the volume adjusted to 3.2 mL with the growth medium. Finally, 250 μL cell suspension was deposited on 12 pre-treated coverslips (18 mm, Amilabo) placed in a 32 mm Petri dish (Falcon®). Coverslips were pretreated with poly-D-lysine (0.5 mg/ml, Sigma-Aldrich®) and laminin (20 μg/ml, Sigma-Aldrich®). After 2–3 hours at 37 °C in a water-saturated atmosphere of 95% O_2_, 5% CO_2_, the volume of growth media was increased to 2 mL for each Petri dish. This pre-plating period allowed cells to attach to the coverslip before the volume was adjusted. Cells were maintained at 37 °C in a water-saturated atmosphere of 95% O_2_, 5% CO_2_ and themedium was changed every 3 days. Ciliary neurotrophic factor (CNTF) at 4 ng/ml (Sigma-Aldrich® or Alomone Laboratories) was added to the culture medium at DIV2 in order to test its incidence on neuronal connectivity. At DIV 6–7 anti-mitotic drugs (cytosine-1-β-D-arabinofuranoside at 1 μM, or uridine/5-fluoro-2′-deoxyuridine mix, 10 μM each) were added to prevent non-neuronal cell proliferation.

### Electrophysiology and analysis

Neurons were easily identified, based on their large size compared to non-neuronal cells (see Fig. 1) and were visualized using infrared differential interference contrast videomicroscopy (Evolve camera, Photometrics®; Olympus BX51WI microscope). Cells were continuously superfused with extracellular solution at 32 °C containing (in mM): 125 NaCl, 2.5 CaCl_2_; 1.5 MgCl_2_; 2.5 KCl; 9.3 HEPES, 7.1 Hepes-Na and 30 glucose, pH 7.4. Voltage and current clamp techniques were used to record synaptic currents and assess neuronal properties. Patch pipettes (3.5–4 MΩ) were pulled from borosilicate glass capillaries (GC150TF-10, Harvard Apparatus, Holliston, MA) on a DMZ-Universal Puller (Zeitz Instruments) and filled with a patch solution containing the following (in mM): 115 K-gluconate, 20 KCl, 10 HEPES, 0.5 EGTA, 2 MgCl_2_, 0.3 CaCl_2_, 0.3 Na-GTP and 2 Na_2_-ATP, pH 7.4, 290–300 mOsm, pH 7.4 (all products were from Sigma-Aldrich®). Analog signals were low-pass filtered at 2.2 kHz (Multiclamp 700B Molecular Devices) and digitized at 20 kHz using a Digidata-1322A interface and pClamp 10 software (Molecular Devices, LLC). Cells were voltage clamped at −60 mV. Cell capacitance and series resistance were not compensated, but continuously monitored using −5 mV test pulses. Only whole cell recordings with access resistance less than 12 MΩ were taken into account for this study and experiments were only analyzed if changes in series resistance were less than 25% of the initial value. In current clamp mode, no holding current was injected and the bridge was balanced.

To measure the properties of action potentials (APs), single spikes were generated by injecting a 20–50 ms pulse of current. AP threshold, peak amplitude and the duration of the AP at half the maximal height of the AP (AP half duration) were measured. For each neuron, measurements were made on at least 6 single APs before averaging. AP threshold was defined as the membrane voltage value reached when the voltage first derivative rises to 5% of its peak amplitude^16^,^30^. Resting membrane potential (RMP) was the average value of the potential measured during a time window (minimal duration of 10 s) with no spontaneous excitatory post synaptic potentials (EPSPs). To determine the input resistance (Rin), 0.5–1 sec hyperpolarizing and depolarizing pulses were used and membrane potentials were measured in the last 100 ms for each pulse. The intensity range of the currents was adjusted for each cell to avoid activation of voltage-dependent conductances. The Rin value was then given by the slope of the voltage vs. current relationship obtained by linear regression.

Excitatory post synaptic currents (EPSCs) were recorded from connected neurons using dual patch-clamp techniques. Single APs were induced in the pre-synaptic cell by brief (20–50 ms) pulses of current every 10 seconds. To determine the basic EPSC properties (latency, peak amplitude, rise and decay time), synaptic responses from 2 minute recordings were averaged following alignment of the presynaptic action potentials using automatic peak detection in clampfit 10 software (Molecular Devices). Latency of the EPSC was defined as the duration between the peak of the AP and the onset of EPSC. Rise and decay times correspond to the time measured between 10 and 90% of EPSC peak amplitude during the rise and decay phases of the EPSC. The variability of the synaptic responses was assessed from the coefficient of variation (CV) of the amplitude of the EPSCs. For each cell, the CV (in %) was calculated as the ratio of the standard deviation to the average amplitude of the EPSC. A protocol of double stimulations was used in order to characterize the paired-pulse ratio (PPR) as PPR can be used as an estimate of the release probability^31^,^32^. PPR (in %) was defined as the amplitude ratio of the second to the first EPSC. Paired-pulse protocols were performed at a frequency of 10–20 Hz depending on the cells. When synaptic responses were monitored over time, only the amplitude of the first EPSCs were considered and plotted against time. A smoothed running average (every two minutes) curve is presented in the plots to facilitate interpretation. EPSC amplitude was normalized to the average value calculated over the first 5 minutes of stimulation or before the arrival of a drug interfering with the synaptic transmission (depending on the experiments). The fluorescence of Alexa fluor® 594 hydrazide sodium salt (30 μM) (Molecular Probes®) was used to monitor presynaptic diffusion of the recording pipette solution containing the interfering peptide L3.4^26^. Fluorescence was monitored over time using an Evolve camera (Photometrics®) piloted with MetaVue interface software (Molecular devices, LLC). Fluorescence excitation was provided by a LED illumination system (X-Cite® 120LED, Lumen Dynamics). Figures were prepared using Sigma Plot, GraphPad Prism 5, Igor Pro, Adobe Photoshop and Illustrator (CS5, Adobe Systems Inc., San Jose, CA, U.S.A.).

### Peptides

Peptides corresponding to the loop linking transmembrane 3 to transmembrane 4 of the ATP6V0c (L3,4: GVRGTAQQPRLF) and its scrambled version (L3.4s: GQATVQPLGRRF) were synthetized by Activotec (Southampton, UK) and used at 2 mM in the recording solution.

## Additional Information

**How to cite this article**: Amendola, J. *et al.* Optimization of neuronal cultures from rat superior cervical ganglia for dual patch recording. *Sci. Rep.* **5**, 14455; doi: 10.1038/srep14455 (2015).

## Figures and Tables

**Table 1:** Electrical properties of DIV14–17 SCG neurons.

	RMP (mV)	input resistance (MΩ)	AP threshold (mV)	AP amp. (mV)	AP half duration (ms)
Mean	–63.0	158.0	–34	69	0.85
Std. Deviation	4.2	57.1	4.9	7.4	0.2
Std. Error	0.8	10.6	0.9	1.4	0.04
Maximum	–54.0	298	–25.9	82.7	1.29
Minimum	–70.0	72.0	–42.6	54.4	0.46
25% percentile	–66.1	114.0	–38.6	63.0	0.71
Median	–63.0	147.0	–34.0	68.1	0.83
75% percentile	–60.0	192.0	–29.3	74.0	1.01
n	29	29	29	29	29

**Table 2:** EPSC properties recorded from connected pair of DIV14–17 cultured SCG neurons.

	Amplitude (pA)	CV (%)	PPR	Latency (ms)	rise time (ms)	decay time (ms)
Mean	267	61.85	2.08	6.7	2.59	14.85
Std. Deviation	310	22.4	1.41	5.16	0.79	5.85
Std. Error	61	4.39	0.31	1.01	0.15	1.28
Maximum	1068	109.6	7.03	19.21	4.66	25.26
Minimum	16	13	0.37	0.86	1.49	4.62
25% percentile	29	46.95	1.09	2.34	2.09	10.07
Median	134	63.2	1.83	5.39	2.4	14.04
75% percentile	449	77.7	2.7	10.6	2.97	20.19
n	26	26	21	26	26	21
